# Eco-friendly pH monitoring in aquaculture: A comparative study of biomass waste extracts with different setup designs

**DOI:** 10.1016/j.mex.2025.103223

**Published:** 2025-02-15

**Authors:** Farhad Nadi, Elham Fazel Najafabadi, Hajar Rastegari

**Affiliations:** a School of Information Technology, UNITAR International University, 47301, Petaling Jaya, Selangor, Malaysia; b Department of Water Engineering, College of Agriculture, Isfahan University of Technology, 84156-83111, Isfahan, Iran; c Institute of Tropical Aquaculture and Fisheries, University Malaysia Terengganu, 21030, Kuala Nerus, Terengganu, Malaysia

**Keywords:** Aquaculture pH monitoring, Smartphone-based colorimetric sensing, Biomass-derived indicators, Mango leaf extract, Polystyrene tray configuration, Eco-friendly pH monitoring in aquaculture

## Abstract

Maintaining optimal water quality is critical for the success of aquaculture operations, where pH monitoring plays a pivotal role. This study presents a novel approach for pH monitoring in aquaculture ponds by harnessing biomass-based indicators and smartphone-based colorimetric sensing using different setups designs. Three biomass indicators including red cabbage, mango leaf, and used coffee grounds extracts were tested. Standard solutions across a pH range of 1–13 were tested using four setups: black and white polypropylene enclosures, a polyethylene pipe assembly, and a polystyrene tray configuration. The polystyrene tray configuration was determined to be the most effective, as its longer light path (4.5 cm) significantly enhanced color visibility and produced more vibrant color changes, making it ideal for further investigations. The method is as follows:

• Water samples were collected from aquaculture ponds. pH were analyzed using this method and standard pH meters.
• Mango leaf extract showed strong pH sensitivity and correlation (R² =0.9654).
• The mango leaf extract attained a quantification accuracy of 0.5 pH units within a pH range of 3–12.
• This smartphone-based approach offers simplicity and ease of implementation empowering aquaculture farmers with a practical tool for monitoring water quality.

## Graphical abstract

**Figure:**
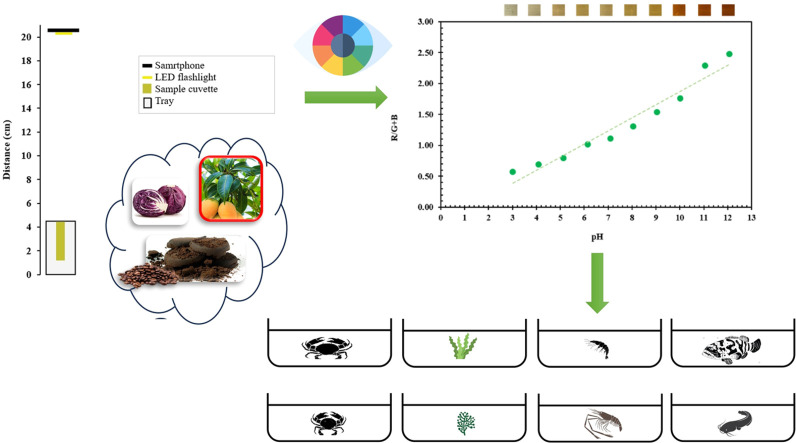

## Introduction

Aquaculture has emerged as a major contributor of global food production, supplying 82.1 million metric tons of seafood in 2018 [32]. This figure is projected to rise significantly, reaching an estimated 202 million metric tons by 2030 [2]. However, the intensification of aquaculture production must be pursued sustainably, incorporating ecologically sound management practices to mitigate its environmental impacts [24]. Among the challenges facing aquaculture, water quality management is paramount, as it directly influences the growth, health, and productivity of aquatic species [13]. Key water quality parameters such as temperature, pH, and dissolved oxygen, play a critical role in nutrient availability, the efficacy of chemical treatments, and the physiological well-being of aquatic organisms [34]. pH is particularly significant, as aquatic species thrive within specific pH ranges, and deviations can adversely affect their growth and survival [3]. The optimal pH range for most aquatic species falls in the range of 5–9.5 [26].

Traditional pH measurement methods, such as automatic titrators and pH meters, are accurate, but costly, requiring skilled operators and regular maintenance [16]. Low-cost alternatives, such as synthetic pH-responsive paper strips, provide only rough estimates, lack accuracy, and pose environmental concerns due to the use of synthetic pH-sensitive reagents [31]. These limitations underscore the need for a cost-effective, simple, and sustainable method for on-field pH determination. In this context, colorimetric pH sensors have gained considerable attention for their applications in environmental monitoring, healthcare diagnostics, food quality assessment, and industrial process control. Their appeal lies in their simplicity, cost-effectiveness, and ability to visually indicate pH changes [11].

Colorimetric reagents derived from biomass have gained attention as eco-conscious substitutes for traditional synthetic chemicals. Their significance lies in reducing dependency on synthetic chemicals. Extraction of these reagents from agricultural waste offers a cost-effective approach for pH sensing, simultaneously tackling waste disposal issues and optimizing resource use. These reagents significantly reduce ecological harm due to biodegradability and non-toxicity. By strategically sourcing and processing biomass, they can be tailored for varied uses, including aquaculture industry, due to their ease of use, even for non-specialists [18].

However, these reagents rely on colorimetric detection that necessitate visual comparison of chromatic variations against standardized reference charts, a process inherently susceptible to subjectivity. To mitigate this limitation, the integration of smartphone technology with colorimetric analysis presents a viable pathway toward developing user-friendly analytical tools. By harnessing smartphone cameras to process optical data in real time, this strategy exploits intrinsic advantages, including portability, rapid data acquisition, and operational simplicity [29]. Such systems facilitate on-site analysis, enabling timely interventions in critical contexts such as water quality monitoring, which is integral to maintaining aquatic ecosystem healthy and productive [6].

Advancements in sustainable smartphone-based sensor design are revolutionizing aquaculture practices. In one study, Haq et al. [12] engineered a paper-based sensor functionalized with anthocyanin extracts from red cabbage, blueberries, and blackberries for rapid ammonia detection. Their protocol, combining solid-liquid extraction with ultrasonication, achieved a detection limit of 2 mg l^-1^ using alkaline red cabbage extract. However, the instability of anthocyanin-based sensors under varying environmental conditions, such as light, temperature, and pH changes, remains a significant challenge. Parallel innovations include a mango leaf extract-immobilized paper sensors developed by Nadi et al. [22], which demonstrated stable ammonia detection in aquaculture effluents with a detection limit of 0.50 mg L⁻¹, a linear dynamic range of 1.70–10.00 mg L⁻¹, and a 45 days sensor stability. Expanding on this, Rastegari et al. [26] incorporated machine learning algorithms into mango leaf extract colorimetric sensors to automate pH classification via image-based colorimetric analysis. Collectively, these advancements provide scalable, real-time solutions for water quality management in resource-limited aquaculture settings. Nevertheless, environmental variables, particularly fluctuating ambient light, remain a barrier to consistent accuracy in smartphone-dependent systems [7,20].

The present study focuses on designing robust, field-ready pH monitoring systems that are suitable for aquaculture applications. Experimental prototypes undergo rigorous stress testing to evaluate durability, while agricultural waste extracts are screened for pH-responsive characteristics. Through iterative optimization of biomass waste extract and systematic comparison of device architectures, the study aims to identify configurations that balance analytical accuracy with practical usability, a critical milestone for advancing sustainable aquaculture practices.

## Materials and methods

### Chemicals and reagents

Ortho-Phosphoric acid (85–88 %) and sodium hydroxide (99 %) were purchased from R & M Chemicals. Ammonium chloride (≥99.5 %), sodium nitrite (≥97 %) and sodium nitrate (≥99.0 %) were purchased from Sigma. All chemicals were of analytical grade and used as received without further purification. Distilled water was used in all experiments. Green mango leaves, used coffee grounds, and red cabbages were sourced from apple mango trees, local coffee shops, and local markets in Kuala Nerus, Terengganu, respectively, during April and June 2024.

### Experimental procedure

Fresh mango leaves were initially rinsed with tap water followed by distilled water to remove surface contaminants. After sequential washing, the leaves underwent drying at 65 °C for 48 h. The dried biomass was mechanically pulverized using a grinder and sieved to obtain a homogeneous powder with particle diameters ≤120 µm. Used coffee grounds underwent identical drying procedure. Aqueous extracts were prepared by refluxing 100 g of dried powder in 500 mL of distilled water at 100 °C for 30 min, followed by centrifugation at 8000 × g for 15 min. Red cabbage extract was prepared using an adapted protocol from Celik, et al.’s [8], wherein 100 g of shredded leaves were boiled in 500 mL of distilled water for 30 min prior to filtration. All extracts were stored at −20 °C for later use.

Standard solutions were prepared through serial dilution of 1 M H_3_PO_4_ and 1 M NaOH stock solutions to achieve precise target pH values in the pH range of 1–13. The dilution ratios were exactly adjusted to achieve the desired pH levels for each standard solution. For colorimetric analysis, 0.5 mL of each extract was added to cuvettes containing 3 mL of standard solutions or aquaculture pond samples. A Samsung A12 smartphone (Seoul, South Korea) was used for imaging. A standard spectrophotometer cuvette with dimensions of 4.5 cm in height, a capacity of 3.5 mL, and an optical range of 190–2500 nm was used as sample container.

**Fig. 1:**
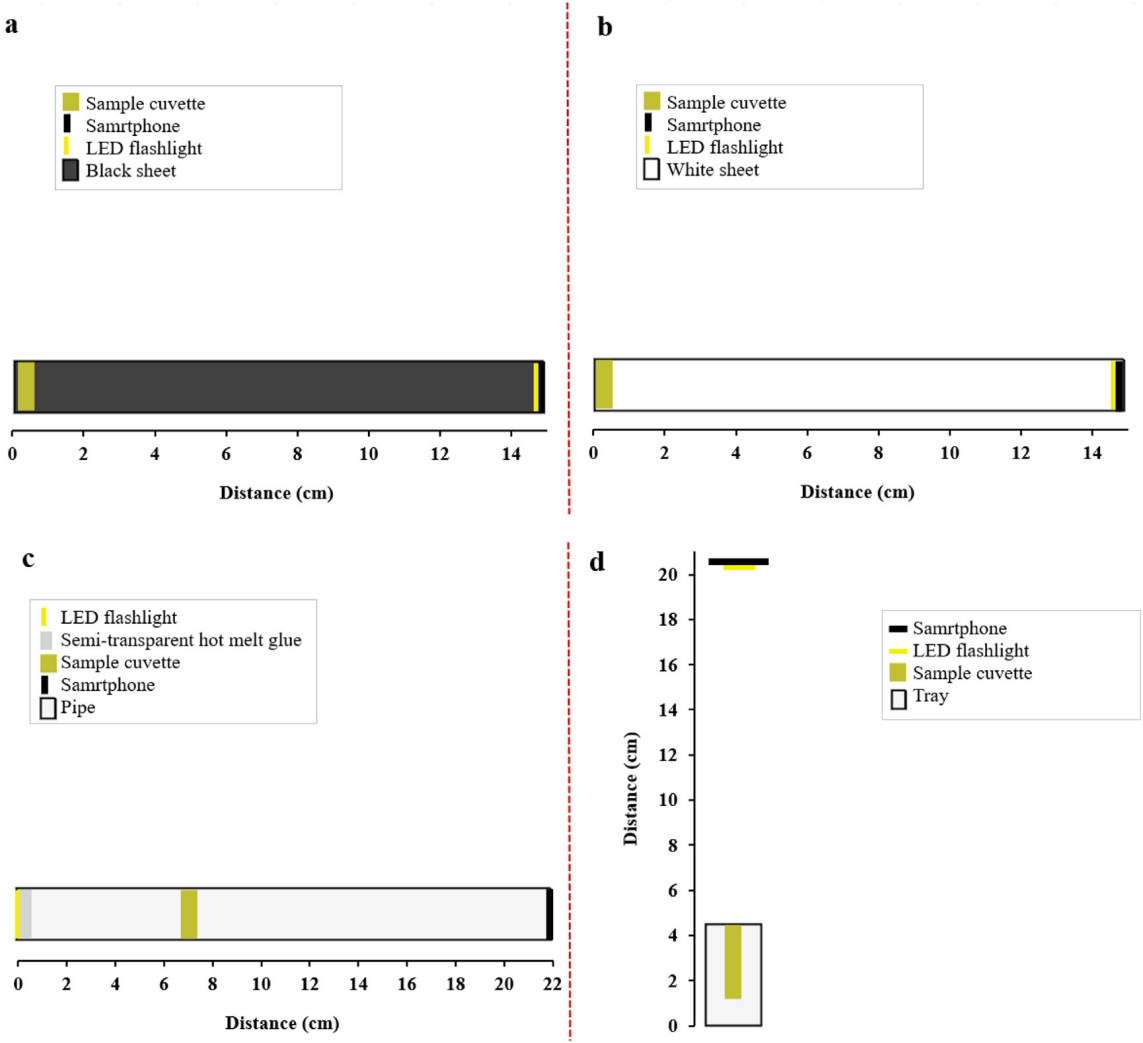
Schematic illustration of four different smartphone colorimetric chambers developed in this study: (a) Black corrugated polypropylene enclosure, (b) White corrugated polypropylene enclosure, (c) Polyethylene pipe assembly, and (d) Tray configuration.

Four distinct photometric chambers were engineered to standardize imaging conditions. These included two designs made from corrugated polypropylene enclosures, one in black (Fig. 1a) and the other in white (Fig. 1b), as well as polyethylene pipe assembly (Fig. 1c) and a polystyrene tray configuration (Fig 1d). Each of these setups was meticulously designed with consideration for the smartphone camera focusing zoom distance, ensuring consistent and reproducible imaging results. corrugated polypropylene enclosures consisted of a structured enclosed box with a width of 15 cm in depth, a height of 5 cm, and featuring a 2 cm triangular cutout as the sample holder. This geometry minimizes lateral light intrusion while maintaining focal consistency.

The pipe assembly (Fig. 1c), consisted of a 22 cm cylindrical chamber with an inner diameter of 4.5 cm, internally coated with 0.5 cm-thick translucent hot-melt adhesive to diffuse light from a proximal Eveready LC1L2A LED source (Uttar Pradesh, India). The smartphone camera was positioned distally, with a lateral sampling port located 7 cm from the light source. In the tray configuration (Fig. 1d), a white foam packaging tray was used as the photometric chamber. This setup enabled top-down imaging at a fixed 15 cm lens-to-cuvette distance, utilizing the smartphone's integrated LED flash for illumination.

### Imaging and image analysis

Following sample placement in the photometric chamber, the smartphone camera acquired images under controlled illumination conditions, yielding 24-bit JPEG files (mean file size: 1.4 MB; resolution: 4000 × 1800 pixels).

A straightforward image processing protocol was implemented to quantify pH-dependent chromatic shifts. Captured images were analyzed using Color Grab (version 3.4.1), a third-party Android application enabling RGB decomposition of spatially resolved color data. Three circular regions of interest (ROIs) were systematically sampled from the designated points of each image to account for spatial heterogeneity. The normalized chromatic index (R/[G + *B*]) was derived from averaged RGB intensity values (0–255 scale) across all ROIs. All measurements were repeated five times under identical experimental conditions and the standard deviation of these measurements was calculated to assess the data variability.

### Accuracy and selectivity assessment of the method

To assess the accuracy of the method, the pH of real samples collected from aquaculture ponds was measured using three pH meters (pH 2700, Eutech; AB15 basic; Fisher brand Accumet). Measurements were repeated five times for each sample, and the standard deviation was calculated to ensure consistency.

The selectivity of the method was tested by introducing common interfering substances found in aquaculture pond water, including ammonia, nitrite, and nitrate. These compounds were selected because they are known to affect water quality and aquatic health. For most aquatic species, safe concentration limits are defined as 2 mg L⁻¹ (ammonia), 0.5 mg L⁻¹ (nitrite), and 50 mg L⁻¹ (nitrate) [23]. Exceeding these levels can induce stress, impair growth, and increase disease susceptibility in aquatic populations [23]. To replicate realistic conditions, the concentration of each interfering substance was set at its maximum permissible limit.

## Results and discussions

### Colorimetric chamber selection

**Fig. 2:**
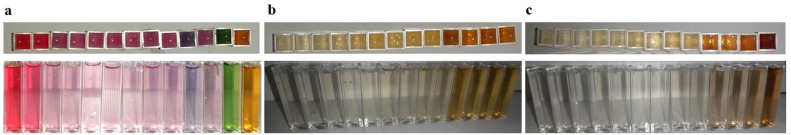
Color intensities in images captured from top-view (up) and side-view (down), featuring (a) red cabbage extract, (b) mango leaf extract, (c) used coffee grounds extract.

In preliminary studies, four setups were developed for colorimetric detection: black corrugated polypropylene enclosure, white corrugated polypropylene enclosure, polyethylene pipe assembly, and tray configuration. As shown in Fig. 2, the tray configuration produced more vibrant colors in images for all samples including red cabbage extract (Fig. 2a), mango leaf extract (Fig. 2b), and used coffee grounds extract (Fig. 2c). This improvement likely stems from the longer optical path length (4.5 cm) in the top-view design compared to the side-view design's shorter path (1 cm). The extended path length enhanced light-sample interaction, amplifying color visibility and intensity. This finding aligns with the reports in the literature, where increased optical path length intensifies color visibility. For example, [19], observed the most distinct color differences in gemstones when thickness (and thus light path) reached 5 mm. Given these findings, the top-view tray configuration was selected for further studies.

### Color change

**Fig. 3:**
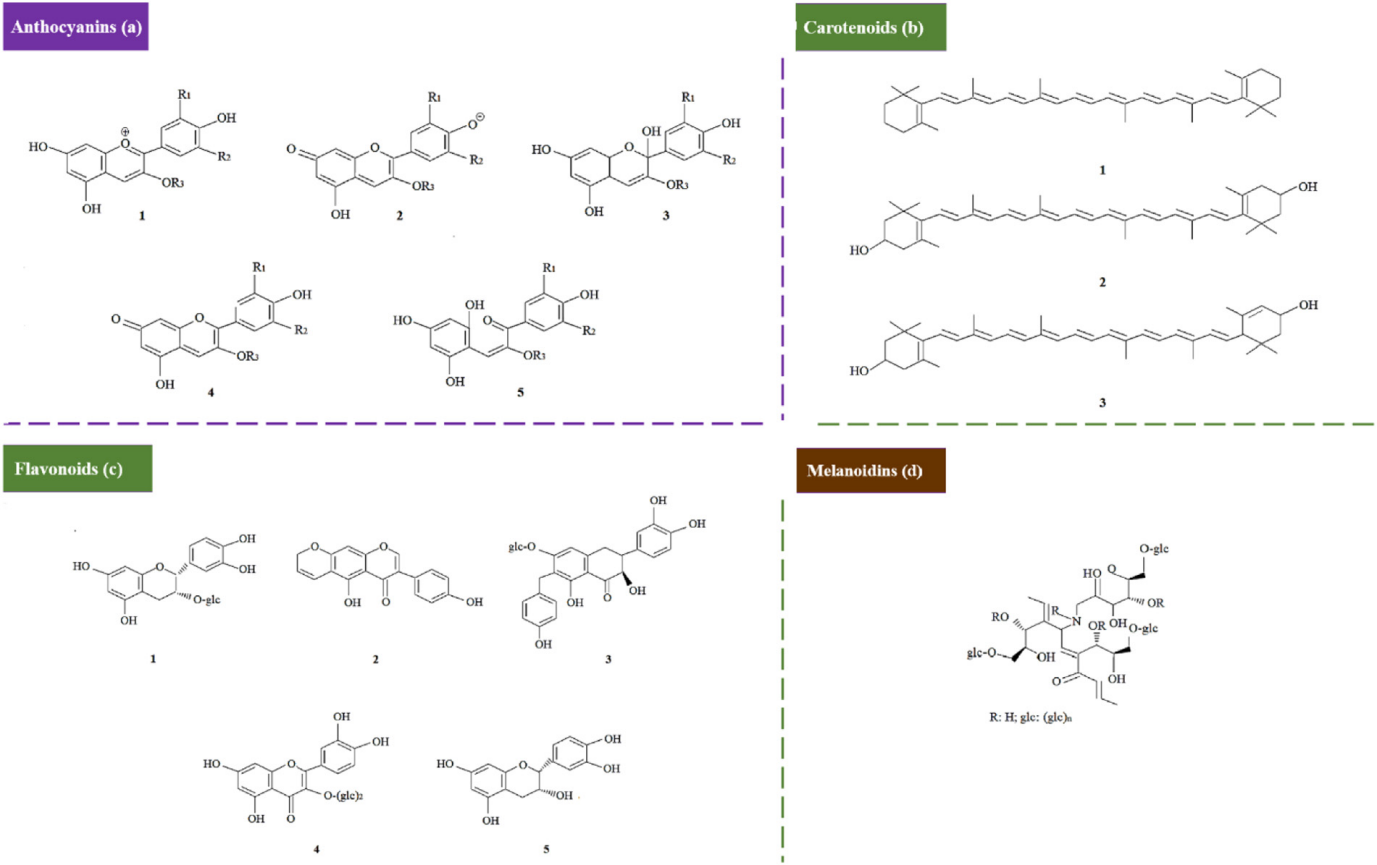
Pigment structures for (a) anthocyanins; 1: flavylium cation, 2: anionic quinoidal base, 3: carbinol pseudo base, 4: quinoidal base, 5: chaleone, [1,17], (b) carotenoids; 1: beta-carotene, 2: zeaxanthin, 3: lutein, [5,14], (c) flavonoids; 1: (-)-epicatechin-3-O-β-glucopyranoside, 2: 5‑hydroxy-3-(4-hydroxylphenyl)pyranone[3,2 g]chromene-4(8H)-one, 3: 6-(p-hydroxybenzyl)taxifolin-7-O-β-d-glucoside, 4: quercetin-3-O-α-glucopyranosyl-(1→2)-β-glucopyranoside, 5: (-)-epicatechin(2-(3,4-dihydroxyphenyl)−3,4-dihydro-2H-chromene-3,5,7-triol) [25], (d) basic structure of melanoidins [21,27].

This study examined three biomass-based colorimetric indicators: red cabbage extract, mango leaf extract, and coffee grounds extract. The color changes observed in these materials arise from pigment structural transformations in response to pH changes [1]. The chemical structures of the key pigments present in red cabbage extract, mango leaf extract, and used coffee grounds extract are shown in Fig. 3.

**Fig. 4:**
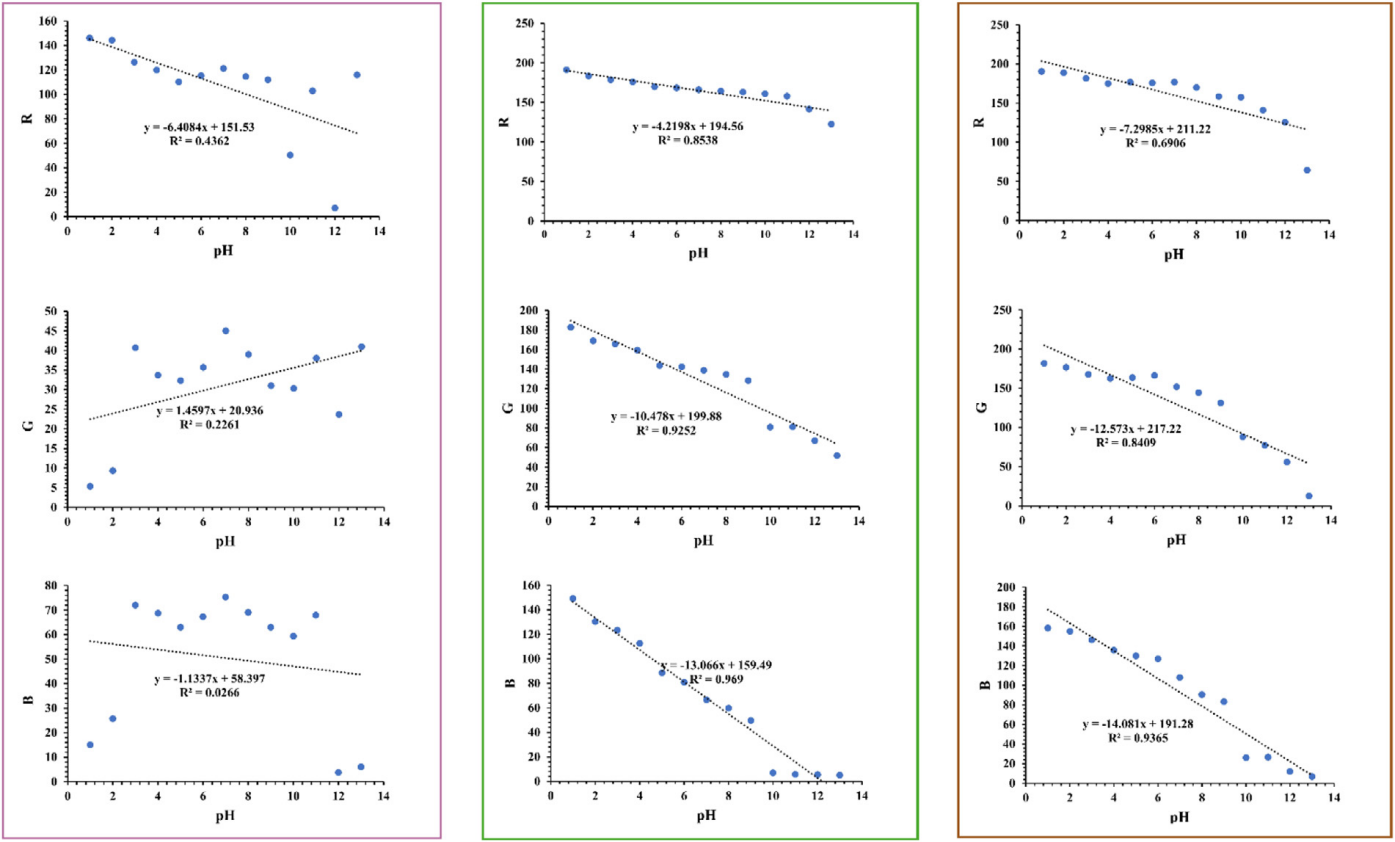
The RGB values extracted from the images of the standard solutions after addition of biomass waste extracts, for pH values in the range of 1–13, (left) red cabbage extract, (middle) mango leaf extract, (right) used coffee grounds extract.

**Fig. 5:**
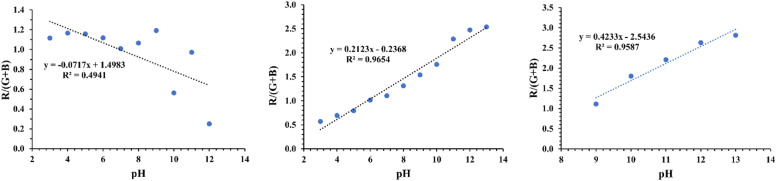
Calibration curves for determination of pH in top-view tray setup with (left) red cabbage extract, (middle) mango leaf powder extract, (right) used coffee grounds extract.

Red cabbage extract contains anthocyanins, a class of hydrophilic pigments (Fig. 3a). Anthocyanins adopt distinct molecular forms depending on the pH. In acidic environments, protonation stabilizes the red-colored flavylium cation (Fig. 3a, 1), causing the solution to appear red [1]. As pH increases to slightly acidic levels, the flavylium cation converts to the colorless carbinol pseudo-base (Fig. 3a, 3), reducing red color intensity. At neutral pH, equilibrium between protonated and deprotonated forms produces a purple hue (Fig. 4a, 4) [17]. Under alkaline conditions, deprotonation generates the blue-shifted quinoidal base (Fig. 3a, 2) shifting the solution's color toward bluish-greens [17]. In highly alkaline environments, anthocyanins degrade into chalcones (Fig. 3a, 5), resulting in a yellow coloration [17].

Mango leaf extract contains carotenoids and flavonoids as its primary pigments [14]. Carotenoids, such as beta-carotene, lutein, and zeaxanthin (Fig. 3b and c), produce yellow to orange hues due to their long conjugated double-bond systems [5]. Flavonoids (Fig. 3c), which are polyphenolic compounds with a flavone backbone along with various functional groups, contribute additional shades of yellow, orange, and red [25]. In mango leaf extract, pH changes alter the protonation and deprotonation of functional groups of these pigments [25]. This ionization affects their water solubility, thereby influencing extraction efficiency and the intensity of observed color changes [25].

Melanoidins, the primary pigments in used coffee grounds, are a complex mixture of hydrophilic polymers containing carbonyl, hydroxyl, and amino functional groups (Fig. 3d). These compounds give used coffee ground extract their characteristic brown color [27]. At elevated pH levels, ionization of melanoidins’ functional groups increases their solubility in water, improving extraction efficiency [21]. Consequently, alkaline conditions enhance the solution's brown coloration due to higher melanoidin concentrations.

### Analytical signal selection

The analytical response determines a method's sensitivity, selectivity, and precision [22]. To evaluate this, RGB values from images of standard solutions, after adding biowaste extracts (Fig. 4), were plotted against pH (1–13). Solutions containing red cabbage, mango leaf, or used coffee grounds extract exhibited distinct color shifts, ranging from yellow and brown to purple, blue, and green.

Red cabbage extract (Fig. 4, left) showed moderate pH sensitivity in the red (R) channel (slope = −6.41) but weak trends in green (G: slope = 1.46) and blue (B: slope = −1.13). The low R² values (0.44, 0.23, and 0.03 for R, G, and B, respectively) indicate poor correlation between RGB intensities and pH. In contrast, mango leaf extract (Fig. 4, middle) demonstrated strong pH sensitivity across all channels, with steeper regression slopes (−4.22, −10.48, and −13.07 for R, G, and B), and high R² values (0.85, 0.92, and 0.97), confirming its reliability as a pH indicator. Similarly, coffee grounds extract (Fig. 4, right) exhibited robust sensitivity, particularly in the B channel (B: slope = −14.08, R² = 0. 94), with R² values improving from moderate (R: slope =−7.30, R²= 0. 69) to high (G: slope =−12.57, R²= 0. 84) across channels.

The analysis in Fig. 4, reveals that individual RGB channels vary in sensitivity and correlation to pH depending on the extract. While mango leaf and used coffee grounds extracts show strong sensitivity and high correlation across all channels, red cabbage extract performs poorly, with its best correlation (0.44) on the R channel. Overlapping trends in the G and B channels further complicate interpretation. To address this, the ratio R/(G + *B*) was calculated by combining the R channel with the sum of G and B channels. This approach amplifies sensitivity differences and strengthens pH correlation (Fig. 5), creating a more reliable parameter for pH monitoring with mango leaf and used coffee grounds extracts [30]. Notably, blank sample RGB values were subtracted from all test samples before signal calculation to minimize background interference.

Red cabbage extract exhibited weaker correlation between the analytical signal and pH (R^2^ =0,49), indicating lower correlation compared to mango leaf and used coffee grounds extracts, which showed strong correlations (R^2^= 0.96, and 0.96, respectively). While red cabbage is widely used as a pH indicator in educational or household settings due to its vivid, accessible color changes, its practicality is limited by the instability of anthocyanins. These pigments degrade rapidly under light or oxidative conditions [15], as the fading of purple/blue hues (pH 8–11) to a uniform pale pink within 2 min was observed. Hence, for accurate pH monitoring, mango leaf and used coffee grounds extracts are more suitable.

The calibration curve for mango leaf extract showed linearity across pH 3–12 (Fig. 5, middle), covering the optimal pH range for most aquatic species (pH 5–9.5). In contrast, used coffee grounds extract had a narrower linear range (pH: 9–13), (Fig. 5, right), limiting its utility in aquaculture practices. Consequently, mango leaf extract is better suited for smartphone-based pH sensors in this context, as evidenced by its adoption in subsequent studies [4,10,33].

### Real sample analysis

To evaluate the method's accuracy, water samples were collected from eight aquaculture ponds at Universiti Malaysia Terengganu's AKUATROP hatchery. Samples were filtered through a 0.22 µm Nylon syringe filter, mixed with mango leaf extract, and imaged using the top-view tray configuration and a smartphone. The pH values were calculated using the calibration curve equation. Table 1 compares the measured pH of real samples with those obtained from the present method, using pH meters as the standard reference.

**Table 1:** Determination of pH using mango leaf extract.

	Sample	This study	pH meter
1	Tap water	6.95 ± 0.18	7.12 ± 0.04
2	Catfish	6.37 ± 0.11	6.75 ± 0.01
3	Giant prawn	6.25 ± 0.13	6.38 ± 0.06
4	Mud crab	8.48 ± 0.28	8.75 ± 0.09
5	Tiger shrimp	7.55 ± 0.32	7.99 ± 0.07
6	Grouper	6.35 ± 0.21	6.68 ± 0.08

The method showed strong agreement with conventional pH meters, yielding an average deviation of 0.5 pH units. These results align with studies using smartphone-based colorimetric pH monitoring. For example, Da-Silva et al. [9] developed an innovative lab-on-a-chip device for water quality monitoring, utilizing colorimetric measurements with a smartphone camera integrated into a microfluidic paper-based analytical device. Their study demonstrated high accuracy in pH measurement, with deviations of <0.6 units when compared to traditional methods such as pH meters [9].

This level of precision is well within acceptable limits for practical pH monitoring, particularly in aquaculture systems, where maintaining water quality does not require extreme accuracy beyond the nearest tenth of a pH unit [26]. This consistency with established literature underscores the reliability of the top-view tray configuration with mango leaf extract for practical pH monitoring in aquaculture systems.

### Interference study

To evaluate the method's selectivity, its performance was tested in the presence of common aquaculture water contaminants including ammonia, nitrite, and nitrate. Standard solutions within the linear pH range (3–12) were spiked with interfering compounds at maximum concentrations of 20 mg L⁻¹ (ammonia), 5 mg L⁻¹ (nitrite), and 500 mg L⁻¹ (nitrate). These were added to standard solutions at a 10 % volume ratio. After mixing with mango leaf extract, images were captured using the smartphone-based top-view tray configuration. The tolerance limit [28], defined as the highest contaminant concentration causing <5 % error in pH determination, was used to assess interference.

As reported in Table 2, nitrite and nitrate had no significant effect on pH measurements. However, ammonia at 2 mg L⁻¹ caused a 5 % error. This concentration aligns with typical ammonia levels in aquaculture systems (1–3 mg L⁻¹) [26]. Despite this interference, the error remains acceptable for practical aquaculture monitoring, where precision to ±0.5 pH units is sufficient for water quality management.

**Table 2:** Results of the study of possible interferences from certain species found in aquaculture ponds in this study.

Species	Effect on pH quantification	Amount tolerated (mg l^-1^)
Ammonia	5 % error	2
Nitrite	–	0.5
Nitrate	–	50

## Conclusions and future trends

This study presents a low-cost, smartphone-based colorimetric method for pH determination in aquaculture using biomass-derived indicators in conjunction with a smartphone-based device. The findings demonstrate that ordinary smartphones are capable of capturing images with sufficient accuracy for pH determination in aquaculture practices. Calibration curves revealed a linear relationship between the analytical signal and pH values, achieving a correlation coefficient of 0.96 for mango leaf extract. To validate the method, pH measurements obtained using the smartphone-based device were compared to those from a standard pH meter. The results indicated strong agreement between the two methods, with an average deviation of <0.5 pH units.

The proposed smartphone-based colorimetric method demonstrates significant potential for pH determination, offering high levels of accuracy and reliability. The portability and ease of use of the device make it particularly suitable for on-site pH measurements, especially in resource-limited environments. Additionally, the cost-effectiveness of the system positions it as an attractive alternative to traditional pH measurement techniques. With further development and optimization, this smartphone-based device has the potential to transform pH monitoring across various applications, providing a portable, affordable, and user-friendly solution for pH determination in diverse settings.

## CRediT authorship contribution statement

**Farhad Nadi:** Conceptualization, Methodology, Writing – review & editing. **Elham Fazel Najafabadi:** Writing – review & editing. **Hajar Rastegari:** Conceptualization, Investigation, Methodology, Writing – original draft, Writing – review & editing.

## Declaration of competing interest

The authors report no declarations of interest.

## Data Availability

The data that has been used is confidential.

## References

[1] Abedi-Firoozjah R., Yousefi S., Heydari M., Seyedfatehi F., Jafarzadeh S., Mohammadi R., Rouhi M., Garavand F. (2022). Application of red cabbage anthocyanins as pH-sensitive pigments in smart food packaging and sensors. Polymers (Basel).

[2] Action S.I. (2020). World fisheries and aquaculture. Food Agric. Organizat..

[3] Alvarado-Ramírez L., Santiesteban-Romero B., Poss G., Sosa-Hernández J.E., Iqbal H.M.N., Parra-Saldívar R., Bonaccorso A.D., Melchor-Martínez E.M. (2023). Sustainable production of biofuels and bioderivatives from aquaculture and marine waste. Front. Chem. Eng..

[4] Anzecc A. (2000). Australian and New Zealand Environment and Conservation Council and Agriculture and Resource Management Council of Australia and New Zealand, Canberra.

[5] Arscott S.A., Tanumihardjo S.A. (2010). Carrots of many colors provide basic nutrition and bioavailable phytochemicals acting as a functional food. Compr. Rev. Food Sci. Food Saf..

[6] Aunsmo A., Persson D., Stormoen M., Romstad S., Jamtøy O., Midtlyng P.J. (2023). Real-time monitoring of cause-specific mortality and losses in industrial salmon farming. Aquaculture.

[7] Bui T.H., Thangavel B., Sharipov M., Chen K., Shin J.H. (2023). Smartphone-based portable bio-chemical sensors: exploring recent advancements. Chemosensors.

[8] Celik C., Demir N.Y., Duman M., Ildiz N., Ocsoy I. (2023). Red cabbage extract-mediated colorimetric sensor for swift, sensitive and economic detection of urease-positive bacteria by naked eye and smartphone platform. Sci. Rep..

[9] Da Silva V.A.O.P., De Freitas R.C., De Oliveira P.R., Moreira R.C., Marcolino-Júnior L.H., Bergamini M.F., Coltro W.K.T., Janegitz B.C. (2020). Microfluidic paper-based device integrated with smartphone for point-of-use colorimetric monitoring of water quality index. Measurement.

[10] Emcr (2006).

[11] Fu M., Yang M., Xu X. (2022). Upconversion fluorescent nanoprobe based on the 4-NP reversible structure for a wide range of pH determination. New J. Chem..

[12] Haq S.U., Aghajamali M., Hassanzadeh H. (2021). Cost-effective and sensitive anthocyanin-based paper sensors for rapid ammonia detection in aqueous solutions. RSC Adv..

[13] Kalaida M., Gordeeva M., Golenishchev-Kutuzov V.A. (2021). E3S Web of Conferences.

[14] Kanwal Q., Hussain I., Siddiqui L.H., Javaid A. (2009). Flavonoids from mango leaves with antibacterial activity. J. Serb. Chem. Soc..

[15] Koop B.L., Soares L.S., Cesca K., Souza V.G.L., Valencia G.A., Monteiro A.R. (2024). Enhancing the stability of anthocyanins extracts through adsorption into nanoclays – development of a smart biohybrid sensor for intelligent food packaging or as natural food additive/preservative. Food Bioprod. Process..

[16] Li H., Li W., McEwan M., Li D., Lian G., Chen T. (2021). Adaptive filtering-based soft sensor method for estimating total nitrogen in aquaponic systems. Comput. Electron. Agric..

[17] Liu D., Zhang C., Pu Y., Chen S., Liu L., Cui Z., Zhong Y. (2022). Recent advances in pH-responsive freshness indicators using natural food colorants to monitor food freshness. Foods.

[18] Liu H., Ding J., Zhang K., Ding L. (2019). Construction of biomass carbon dots based fluorescence sensors and their applications in chemical and biological analysis. TrAC Trends Anal. Chem..

[19] Liu W., Qiu Y., Guo Y. (2024). Mineralogical characteristics of color-changing garnet and the effect of light path length on color. Sci. Adv. Mater..

[20] Malik S., Singh J., Saini K., Chaudhary V., Umar A., Ibrahim A.A., Akbar S., Baskoutas S. (2024). Paper-based sensors: affordable, versatile, and emerging analyte detection platforms. Anal. Methods.

[21] Mediani A., Kamal N., Lee S.Y., Abas F., Farag M.A. (2023). Green extraction methods for isolation of bioactive substances from coffee seed and spent. Sep. Purif. Rev..

[22] Nadi F., Hossain S., Rahmat R.F., Kasan N.A., Ikhwanuddin M., Rastegari H. (2024). Detection of ammonia in aquaculture wastewater using mango leaf extract-immobilized paper sensors and smartphone colorimetric analysis. Microchem. J..

[23] Nagaraju T.V., BM S., Chaudhary B., Prasad C.D., R G. (2023). Prediction of ammonia contaminants in the aquaculture ponds using soft computing coupled with wavelet analysis. Environ. Pollut..

[24] Nisar U., Peng D., Mu Y., Sun Y. (2022). A Solution for sustainable utilization of aquaculture waste: a comprehensive review of biofloc technology and aquamimicry. Front. Nutr..

[25] Pan J., Yi X., Zhang S., Cheng J., Wang Y., Liu C., He X. (2018). Bioactive phenolics from mango leaves (Mangifera indica L.). Ind. Crops Prod..

[26] Rastegari H., Rahmat R.F., Nadi F. (2024). A machine learning approach to pH monitoring: mango leaf colorimetry in aquaculture. Int. J. Adv. Comput. Sci. Appl..

[27] Santal A.R., Singh N. (2013). Biodegradation of melanoidin from distillery effluent: role of microbes and their potential enzymes. Biodegrad. Hazard. Spec. Prod..

[28] Shahvar A., Shamsaei D., Saraji M. (2020). A portable smartphone-based colorimetric sensor for rapid determination of water content in ethanol. Measurement.

[29] Silva E., M G., Garcia J.A., Garitta D.E., Alencar J., Cunha F., D G., Finkler N.R., Mendiondo E.M., Ghiglieno F. (2022). Smartphone-based spectrometry system as a prescreening assessment of copper and iron for real time control of water pollution. J. Environ. Manage..

[30] Soares S., Fernandes G.M., Rocha F.R.P. (2023). Smartphone-based digital images in analytical chemistry: why, when, and how to use. TrAC Trends Anal. Chem..

[31] Sruthi P.S., Balasubramanian S., Kumar P.S., Kapoor A., Ponnuchamy M., Jacob M.M., Prabhakar S. (2021). Eco-friendly pH detecting paper-based analytical device: towards process intensification. Anal. Chim. Acta.

[32] Steenson S., Creedon A. (2022). Plenty more fish in the sea?–is there a place for seafood within a healthier and more sustainable diet?. Nutrition Bulletin.

[33] Stone N.M., Thomforde H.K. (2004).

[34] Vargas-Muñoz M., Morales J., Cerdà V., Ferrer L., Palacio E. (2023). Paper sensor-based method using a porTable 3D-printed platform and smartphone-assisted colorimetric detection for ammonia and sulfide monitoring in anaerobic digesters and wastewater. Microchem. J..

